# Supplementary material for: Antidepressant Medication Does Not Contribute to the Elevated Circulating Concentrations of Acylethanolamides Found in Substance Use Disorder Patients
Source: Int J Mol Sci. 2023 Sep 30;24(19):14788. doi: 10.3390/ijms241914788 (PMC10573451; doi:10.3390/ijms241914788)
Supplement: Supplementary file 1 [file ijms-24-14788-s001.zip › ijms-2588255-supplementary.pdf]

SUPPLEMENTARY MATERIALS

Table S1: Baseline sociodemographic characteristics of Control and SUD populations

Variable		**CONTROL (N=175)**	**SUD (N=333)**	p value
Sex [n (%)]	**Women**	80 (44.4)	67 (20.1)	**<0.001[a]**
	**Men**	95 (52.8)	266 (79.6)	
Age	median (IQR)	40.0 (31.5-47.0)	43.0 (34.4-51.0)	**0.005[b]**
BMI	median (IQR)	24.8 (22.31-27.0)	25.56 (22.99-29.05)	**0.001[b]**
Marital status [n (%)]	**Single**	82 (45.6)	116 (34.8)	**<0.001[a]**
	**Married/cohabiting**	59 (32.8)	131 (39.3)	
	**Divorced/separated**	13 (7.2)	80 (24.0)	
	**Widowed**	2 (1.1)	6 (1.8)	
Education [n (%)]	**≤ Primary**	7 (3.9)	116 (34.8)	**<0.001[a]**
	**Secondary**	55 (30.6)	170 (51.1)	
	**University**	102 (56.6)	47 (14.1)	
Work status [n (%)]	**Employed**	136 (75.5)	101 (30.3)	**0.001[a]**
	**Unemployed**	23 (12.8)	186 (55.8)	
	**Sickleave**	2 (1.1)	37 (11.1)	
	**Housework**	2 (1.1)	9 (2.7)	
Psychiatric comorbidity [*n* (%)]	**Mood Disorders**	-	143 (42.9)	-
	**Anxiety Disorders**	-	95 (28.5)	
	**Psychotic Disorders**	-	36 (10.8)	
	**Personality Disorders**	-	85 (25.5)	
	**ADHD**	-	33 (9.9)	
	**>2 psychiatric disorders**	-	232 (69.7)	
Substance use disorders [*n* (%)]	**Alcohol**	-	281 (84.4)	-
	**Cocaine**	-	201 (60.4)	-
	**Cannabis**	-	78 (23.4)	-
	**>2 substances**	-	178 (53.5)	-
Psychotropic medication [*n* (%)]	**Antidepressants**	-	132 (39.6)	-
	**Anxiolytics**	-	155 (46.5)	-

	**Antipsychotics**	-	40 (12)	-
	**Disulfiram**	-	133 (39.9)	-
SUD duration years [median (IQR)]	**AUD**	-	10 (4–17)	-
	**CUD**	-	5 (2–12)	-
Days of abstinence [median (IQR)]	**AUD**	-	69 (2–210)	-
	**CUD**	-	25 (0.75–120)	-

([a]) *p*-value from chi-square test; ([b]) *p*-value from Mann–Whitney U test. *p*-value in bold indicates a statistically significant difference. Abbreviations: BMI = body mass index; IQR = interquartile range; SD = standard deviation; SUD = substance use disorder; AUD = alcohol use disorder; CUD: cocaine use disorder.

Table S2: Plasma concentrations of acylethanolamides in both, control and SUD population **(data published previously:** Herrera-Imbroda J, Flores-López M, Requena-Ocaña N, et al (2023a) Antipsychotic Medication Influences the Discriminative Value of Acylethanolamides as Biomarkers of Substance Use Disorder. International Journal of Molecular Sciences 2023, Vol 24, Page 9371 24:9371. https://doi.org/10.3390/IJMS24119371)

**NAEs**	**Control** (N=180)	**SUD** (N=333)	**U-Statistic**	**P value**
**AEA** median (IQR)	0.33 (0.21-.048)	0.44 (0.30-0.64)	20219.50	**.000**
**DEA** median (IQR)	0.08 (0.05-0.14)	0.13 (0.09-0.18)	8207.50	**.000**
**DGLEA** median (IQR)	0.07 (0.04-0.09)	0.08 (0.06-0.12)	20123.00	**.000**
**DHEA** median (IQR)	0.42 (0.27-0.61)	0.52 (0.37-0.72)	20054.50	**.000**
**LEA** median (IQR)	0.70 (0.51-0.93)	1.08 (0.86-1.38)	11351.00	**.000**
**OEA** median (IQR)	1.88 (1.33-3.15)	3.37 (2.52-4.51)	11951.50	**.000**
**PEA** median (IQR)	1.59 (1.27-2.45)	3.37 (2.25-5.72)	9070.00	**.000**
**POEA** median (IQR)	0.29 (0.16-0.39)	0.31 (0.19-0.43)	4752.00	.276
**SEA** median (IQR)	4.30 (0.90-5.90)	2.06 (1.30-4.20)	18817.50	.775

p-value from Mann-Whitney U test. *P*-value in bold indicates a statistically significant difference.

TABLE S3 Baseline sociodemographic characteristics of Alcohol and Cocaine populations

VARIABLE		Alcohol (N = 182)	Cocaine (N = 151)	P value
SEX [N (%)]	**Men**	131 (72)	135 (89.4)	**<0.001**[a]
	**Women**	51 (28)	16 (10.6)	
AGE (mean ± SD)		49 ± 9	37 ± 10	**<0.001**[b]
BMI (mean ± SD)		26.17 ± 4.49	26.58 ± 5.28	0.443[b]
Psychiatric comorbidity [N (%)]	**Mood Disorders**	80 (44)	63 (41.7)	0.682[a]
	**Anxiety Disorders**	50 (27.5)	45 (29.8)	0.639[a]
	**Psychotic Disorders**	15 (8.2)	21 (13.9)	0.097[a]
	**Personality Disorders**	37 (20.3)	48 (31.8)	**0.017**[a]
	**ADHD**	47 (25.8)	19 (12.6)	**0.003**[a]
	**>2psychiatric disorders**	124 (68.1)	108 (71.5)	0.503[a]
Substance use disorders [N (%)]	**Alcohol**	181 (99.5)	100 (66.2)	**<0.001**[a]
	**Cocaine**	57 (31.3)	144 (95.4)	**<0.001**[a]
	**Cannabis**	24 (13.2)	54 (35.8)	**<0.001**[a]
	**>2 substances**	72 (39.6)	106 (70.2)	**<0.001**[a]
Psychotropic medication [N (%)]	**Antidepressants**	74 (40.7)	58 (38.4)	0.676[a]
	**Anxiolytics**	76 (41.7)	80 (53)	0.073[a]
	**Antipsychotics**	15 (8.2)	25 (16.6)	**0.020**[a]
	**Disulfiram**	115 (63.2)	18 (11.9)	**<0.001**[a]

([a]) *p*-value from chi-square test; ([b]) *p*-value from T Student test. *p*-value in bold indicates a statistically significant difference. Abbreviations: BMI = body mass index; SD = standard deviation.

TABLE S4 Plasma concentrations of acylethanolamides according to control group, SUD patients (without antidepressant) and SUD + antidepressant patients.

**ECbs**	**Control (N=175)**	**SUD** (N=201)	**SUD + Antidepressant** (N=132)	**P value**
**AEA** median (IQR)	0.33 (0.21-0.48)	0.44 (0.30-0.64)	0.44 (0.31-0.65)	**<0.001**
**DEA** median (IQR)	0.08 (0.05-0.14)	0.12 (0.09-0.16)	0.14 (0.10-0.19)	**<0.001**
**DGLEA** median (IQR)	0.07 (0.04-0.09)	0.08 (0.06-0.13)	0.08 (0.06-0.12)	**<0.001**
**DHEA** median (IQR)	0.42 (0.27-0.61)	0,54 (0,36-0,74)	0.51 (0.38-0.70)	**<0.001**
**LEA** median (IQR)	0.70 (0.51-0.93)	1.07 (0,86-1.35)	1.12 (0.86-1.42)	**<0.001**
**OEA** median (IQR)	1.88 (1.33-3.15)	3.26 (2.47-4.26)	3.66 (5.59-4.76)	**<0.001**
**PEA** median (IQR)	1.59 (1.27-2.45)	3.02 (2.22-4.96)	4.12 (2.38-6.60)	**<0.001**
**POEA** median (IQR)	0.29 (0.16-0.39)	0.27 (0.18-0.42)	0.33 (0.23-0.50)	0.087
**SEA** median (IQR)	4.30 (0.90-5.90)	2.07 (1.21-4.22)	1.97 (1.47-4.15)	0.893

P-value from Kruskal-Wallis test. *P*-value in bold indicates a statistically significant difference.

TABLE S5 Logistic regression model for distinguishing patients with SUD

Table S5. Logistic regression model for distinguishing patients with SUD

Variable	B	S.E.	Wald	df	p-value	Exp(B)	I.C. 95% para EXP(B)	
							Inferior	Superior
Sex	1,209	,603	4,019	1	,045	3,349	1,027	10,914
Age	-,017	,023	,553	1	,457	,983	,940	1,028
BMI	-,021	,062	,121	1	,728	,979	,867	1,104
AEA[(a)]	7,708	2,768	7,753	1	,005	2225,302	9,798	505429,601
DEA[(a)]	-3,638	1,735	4,399	1	,036	,026	,001	,788
DGLEA[(a)]	1,456	2,458	,351	1	,554	4,289	,035	530,219
DHEA[(a)]	-4,217	1,722	5,996	1	,014	,015	,001	,431
LEA[(a)]	-,257	2,622	,010	1	,922	,773	,005	131,766
OEA[(a)]	1,346	3,261	,170	1	,680	3,842	,006	2293,625
PEA[(a)]	5,256	2,365	4,937	1	,026	191,634	1,859	19758,811
POEA[(a)]	-1,049	1,084	,937	1	,333	,350	,042	2,931
SEA[(a)]	-8,782	2,029	18,737	1	,000	,000	,000	,008
Constant	-,299	4,286	,005	1	,944	,742		

[(a)]Log10 values
Abbreviations: B=Coefficient; BMI=Bodymassindex; CI=Confidenceinterval; df=Degrees of freedom; SE=Standard error; W=Wald test.

TABLE S6

**Table S6.** Logistic regression model for distinguishing SUD patients from SUD with antidepressant treatment patients								
Variable	B	S.E	Wald	df	p-value	Exp(B)	I.C. 95% para EXP(B)	
							Inferior	Superior
Sex	-1.632	0.543	9.049	1	0.003	0.195	0.067	0.566
Age	-0.030	0.020	2.341	1	0.126	0.970	0.933	1.009
BMI	0.076	0.044	2.983	1	0.084	1.079	0.990	1.177
AEA[(a)]	3.225	3.190	1.022	1	0.312	25.163	0.048	13068.958
DEA[(a)]	1.140	1.882	0.367	1	0.545	3.125	0.078	124.942
DGLEA[(a)]	1.766	2.042	0.748	1	0.387	5.847	0.107	319.873
DHEA[(a)]	-0.668	1.437	0.216	1	0.642	0.512	0.031	8.563
LEA[(a)]	-1.591	2.048	0.604	1	0.437	0.204	0.004	11.280
OEA[(a)]	-3.114	3.556	0.767	1	0.381	0.044	0.000	47.234
PEA[(a)]	-0.828	1.900	0.190	1	0.663	0.437	0.011	18.105
POEA[(a)]	0.452	1.091	0.172	1	0.678	1.572	0.185	13.332
SEA[(a)]	3.030	1.736	3.044	1	0.081	20.692	0.688	622.157
Constant	6.166	4.617	1.784	1	0.182	476.453		

[(a)]Log10 values
Abbreviations: B=Coefficient; BMI=Bodymassindex; CI=Confidenceinterval; df=Degrees of freedom; SE=Standard error; W=Wald test.

Figure S1

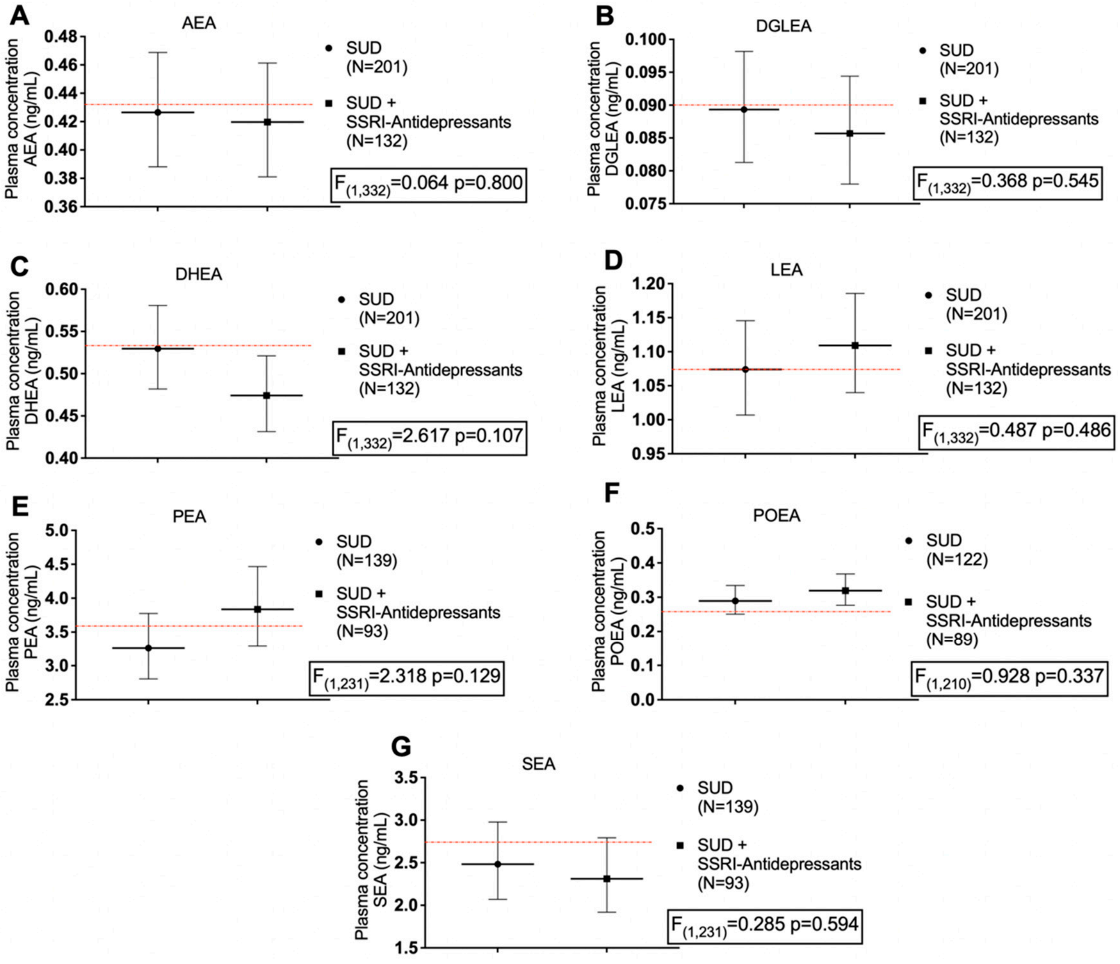

Figure S1. Plasma concentrations of (**A**) Arachidonoyl-ethanolamide (AEA), (**B**) dihomo-γ-linolenylethanolamide (DGLEA), (**C**) Docosahexaenoylethanolamide (DHEA), (**D**) linoleoylethanolamide (LEA), (**E**) Palmitoylethanolamide (PEA), (**F**) Palmitoleoylethanolamide (POEA), and (**G**) Stearoyl-ethanolamide (SEA) in patients with Substance Use Disorder (SUD) not using antidepressants, and patients of SUD using antidepressants. Red line represents the mean plasma concentration of the control healthy population. Data were analyzed by one-way analysis of covariance (ANCOVA). Bars are estimated marginal means and 95% confidence intervals.

Figure S2.

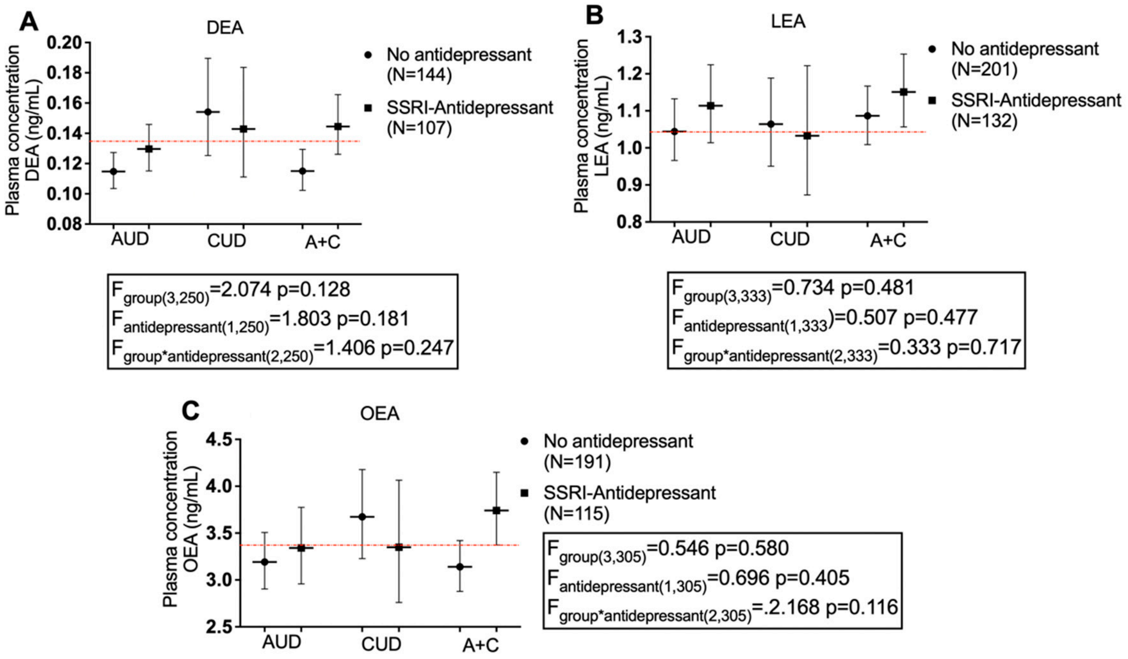

Figure S2. Plasma concentrations of (**A)** Docosatetraenoyl-ethanolamide (DEA), (**B)** Linoleoylethanolamide (LEA), and (**C)** oleoylethanolamide (OEA) in patients with Substance Use Disorder (SUD) not using antidepressants, and patients of SUD using antidepressants classified on the basis of their diagnosis of alcohol use disorder (AUD), cocaine use disorder (CUD) or AUD + CUD. Red line represents the mean plasma concentration of the control healthy population. Data were analyzed by two-way analysis of covariance (ANCOVA). Bars are estimated marginal means and 95% confidence intervals.

Figure S3:

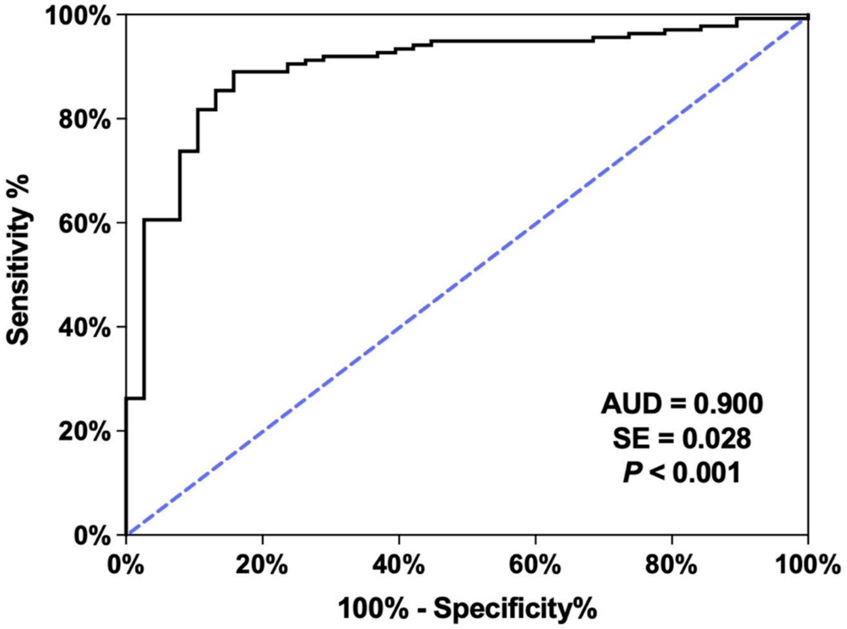

Figure S3. ROC analyses for plasma concentrations of acylethanolamides-based multivariate full models of predictive analysis of discrimination of control population from SUD patients. From reference Herrera-Imbroda J, Flores-López M, Requena-Ocaña N, et al (2023a) Antipsychotic Medication Influences the Discriminative Value of Acylethanolamides as Biomarkers of Substance Use Disorder. International Journal of Molecular Sciences 2023, Vol 24, Page 9371 24:9371. https://doi.org/10.3390/IJMS24119371
